# A Versatile Elastin‐Like Carrier for Bioactive Antimicrobial Peptide Production and Delivery

**DOI:** 10.1002/mabi.202300236

**Published:** 2023-09-24

**Authors:** Laura Colomina – Alfaro, Paola Sist, Silvia Marchesan, Ranieri Urbani, Artemis Stamboulis, Antonella Bandiera

**Affiliations:** ^1^ Department of Life Sciences University of Trieste via L. Giorgieri, 1 Trieste 34127 Italy; ^2^ Department of Chemical and Pharmaceutical Sciences University of Trieste via L. Giorgieri, 1 Trieste 34127 Italy; ^3^ School of Metallurgy and Materials Biomaterials Research Group University of Birmingham Edgbaston Birmingham B15 2TT UK

**Keywords:** antimicrobial peptides, hydrogel matrix, recombinant biopolymers, smart materials

## Abstract

Elastin‐like polypeptides are biotechnological protein and peptide carriers that offer a vast scope of applicability. This work aims to build a model for the expression of antimicrobial peptides (AMPs) by genetically engineering the Human Elastin‐like Polypeptide platform developed in the lab. The well‐characterized AMP indolicidin is selected as an example of an antimicrobial domain for the recombinant fusion at the C‐terminus of the carrier. The fusion construct has been designed to allow the release of the antimicrobial domain. The expression product has been purified and its physicochemical and antimicrobial properties has been characterized. Taking advantage of the self‐assembling and matrix‐forming properties of the recombinant biopolymer, the materials that are obtained have been evaluated for antimicrobial activity toward bacterial‐strain models. This approach represents a cost‐effective strategy for the production of smart components and materials endowed with antimicrobial capacity triggered by external stimuli.

## Graphical abstract

The design, production, and characterization of a model fusion construct between an elastin‐like polypeptide carrier and the antimicrobial domain indolicidin is described. Notably, the fusion protein maintains both the thermo‐responsive behavior of the carrier and the antimicrobial activity of the added bioactive domain. This route of production allows antimicrobial peptide purification as well as the realization of bioactive antimicrobial surfaces that inhibit bacterial growth.

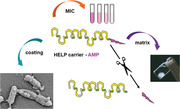

## 1 Introduction

The issue of antimicrobial resistance is considered a major threat to human health and new strategies as well as multidisciplinary approaches are necessary to face this global emergency.^[^ ^1^, ^2^ ^]^ The host defense peptides, which are part of the innate immune response and are widespread throughout all living organisms, constitute the first barrier against attack by pathogens. These naturally occurring peptides, also known as antimicrobial peptides (AMPs), were selected by evolution and have recently raised great interest as a promising alternative to synthetic antibiotics.^[^ ^3^ ^]^

They have broad‐spectrum activity against multiple types of microorganisms, yet there are several limitations to their employment as drug candidates, mainly related to their stability, solubility, cytotoxicity, and bioavailability.^[^ ^4^ ^]^ One strategy to improve the delivery of these biodrugs is the conjugation with polymers that may extend their half‐life, protect them from degradation, and increase their solubility.^[^ ^5^, ^6^, ^7^ ^]^ The recombinant fusion approach offers several advantages, being a cost‐effective means for large‐scale peptide manufacturing. Among the described fusion partners, the elastin‐like polypeptides (ELPs) carriers are still underexplored and offer a wide scope in terms of applicability.

ELPs are protein‐based biopolymers inspired from mammalian tropoelastin. They can be chemically synthesized, however, the expression of recombinant products coded by easily customizable synthetic genes is preferred to overcome chain length limitations. They are generally comprised of repetitive VPGXG motifs, X being any amino acid except proline, which characterizes the bovine homolog.^[^ ^8^ ^]^ They display a reversible thermo‐responsive behavior, similar to tropoelastin, that renders them promising candidates for the development of advanced materials for controlled drug delivery, tissue engineering, and regenerative medicine.^[^ ^9^ ^]^

Up to now, roughly twenty ELP fusion constructs with AMPs have been described, and recently reviewed.^[^ ^10^ ^]^ These ELP carriers are substantially based on the artificial repetitions of the canonical pentapeptidic motif. In our lab, synthetic genes coding for ELPs inspired by the sequences of the human homolog and thus named HELPs (Human Elastin‐like Polypeptides) were assembled and expressed in *Escherichia coli*.^[^ ^11^ ^]^ Compared to the other described ELPs, our HELP biopolymer contains not only the hydrophobic elastin‐like sequences, but also the elastin‐derived cross‐linking domains, mimicking the human tropoelastin structure.^[^ ^12^ ^]^ This feature enabled the setup of an enzymatic method to cross‐link the biopolymer chains to yield a stable hydrogel matrix.^[^ ^13^ ^]^ Thus, our HELP carrier is a multifunctional fusion partner that strategically displays thermo‐responsive behavior with dual function. First, it simplifies the purification of the expression product, and second it endows the resulting matrix with responsivity to release the bioactive motif upon proteolytic stimuli.

The aim of the work described in this paper is to apply our technology to produce AMPs therefore establishing a model for bioactive AMP production. Indolicidin (In) is a thirteen amino acids long cationic peptide belonging to the cathelicidin family, first isolated from bovine neutrophils.^[^ ^14^ ^]^ Showing a broad spectrum of biological activity against a wide range of targets, it was often chosen as a model.^[^ ^15^ ^]^ We selected this AMP as the antimicrobial domain and the fusion partner of HELP. The synthetic gene was specifically tailored, introducing some functional elements like the proteolytic cleavage site and the linker, thus giving an example of the broad range of customization options that our platform offers.

We report the new construct expression, purification, and characterization of its biochemical properties as well as the realization of the derived matrix and the evaluation of the antimicrobial activity toward *Staphylococcus aureus, Pseudomonas aeruginosa* and *Escherichia coli* strains.

## 2 Results and Discussion

### 2.1 Antimicrobial Help Fusion Construct Production and Characterization

**Figure 1:**
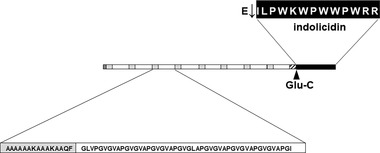
Schematic representation of the HIn construct structure. The theoretical molecular mass is 49 kDa. Dark grey, His‐tag; hatched, linker; black, In domain; light grey, cross‐linking domains; white, elastin‐like hexapeptidic repeats.

Indolicidin (In) is a well‐described and characterized 13‐residue cationic AMP that is often selected as a model peptide, since it has a broad spectrum of biological activity, and is effective against a wide range of bacteria.^[^ ^15^, ^16^ ^]^ Moreover, its high tryptophan content confers it with fluorescence, facilitating its localization. We selected this AMP as the bioactive fusion domain with HELP for these reasons. In **Figure** **1**, the schematic structure of this new construct named HIn is shown, and a short linker region was placed between HELP and the In domain. We decided to insert a glutamic acid residue just before the In sequence to obtain a unique specific proteolytic cleavage site to release the AMP, since neither In nor HELP possess this amino acid in their sequence. However, different specific proteolytic sites, like those for the TEV protease and factor Xa, rather than amino acids for specific chemical cleavage as well as linkers, spacers, and other functional sequences may be introduced depending on the requirements of the desired application.

**Figure 2:**
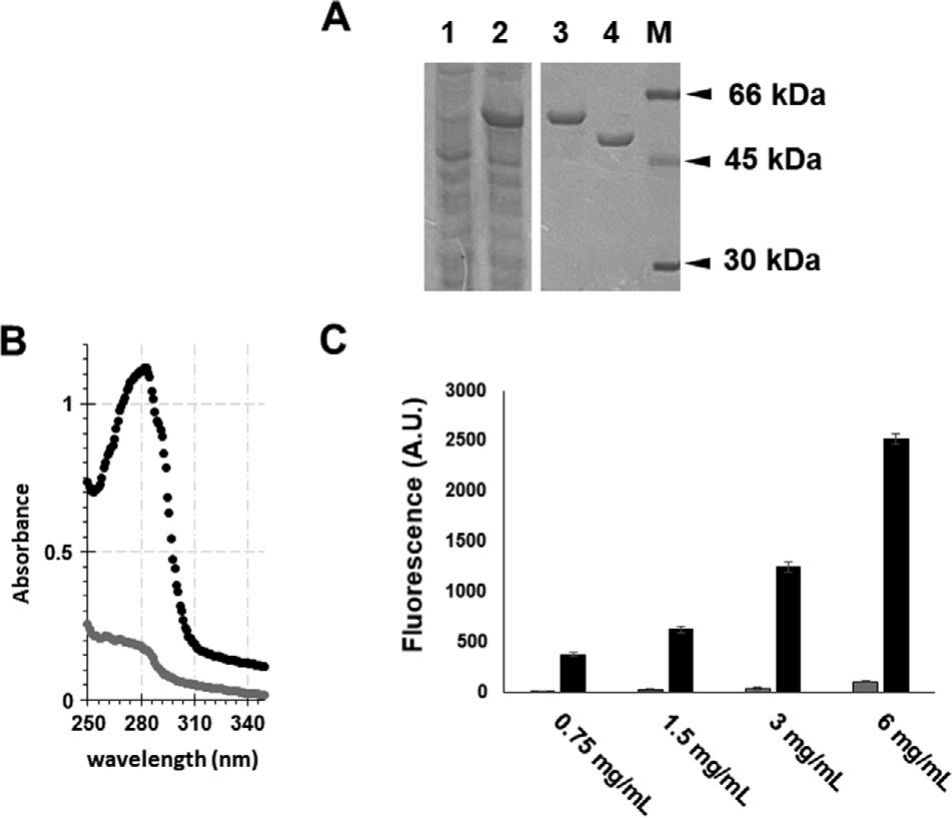
Production and characterization of the HIn construct. A) Representative SDS‐PAGE analysis of the HIn construct. Lane 1, total protein content of the bacterial lysate before IPTG induction; lane 2, total protein content of the bacterial lysate 5 h after IPTG induction; lane 3, HIn construct purified by inverse phase transition cycling; lane 4, purified HELP biopolymer. Molecular masses of the protein markers (M) expressed in kilodaltons (kDa), are bovine serum albumin (66 kDa), ovalbumin (45 kDa), carbonic anhydrase (30 kDa). Coomassie blue staining. B) Absorbance spectra of HELP (grey) and HIn (black) biopolymers. C) Spectrofluorimetric analysis of aqueous solutions of different concentrations of HELP (grey bars) and HIn (black bars). The values represented the mean ± SD, *n* = 4.

HIn was expressed on a lab scale with a high efficiency of the purified product obtained by exploiting the reversible thermo‐responsive behavior that characterizes HELP and that was maintained by this construct (**Figure** **2**A and Supporting Information). Due to the presence of tryptophan in the antimicrobial domain, the absorbance spectrum of HIn showed a peak centered at 280 nm, that is considerably reduced in HELP (Figure 2B). This was confirmed by the fluorescence analysis, where HIn showed clear dose‐dependent signals relative to HELP, which did not show such behavior, as expected (Figure 2C).

**Figure 3:**
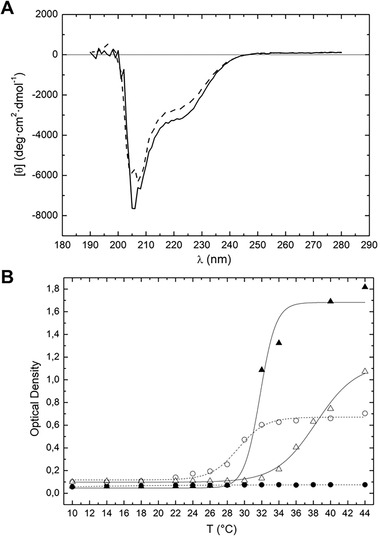
Physicochemical characterization of the HIn biopolymer. A) CD spectra recorded at 25 °C for HIn (black line) and compared with that of the HELP biopolymer (dashed line) in NaPi buffer solution at 0.1 mg mL^−1^. B. Optical density profiles of 2 mg mL^−1^ solutions of HIn in NaPi buffer (black circle) and NaPi/0.15 M NaCl buffer (black triangle) solution as a function of temperature. HELP behavior in NaPi buffer (open circle) and in NaPi/0.15 M NaCl buffer (open triangle) is also reported for comparison.

**Table** **1** reports the features of the secondary structure of HELP and HIn biopolymers obtained with the Expasy tools.^[^ ^17^ ^]^ The physicochemical parameters for the biopolymers were calculated from their primary structure. Only a slight difference in the hydropathy index (Table 1) between the two biopolymers was predicted. In the same table, the results of the deconvolution of the CD spectra of **Figure** **3**A for HIn and HELP are shown, and they are consistent with the theoretical predictions. The CD spectra of HELP and HIn did not show any significant change when 0.15 M NaCl was added to both biopolymer solutions (Figure S1, Supporting Information). However, despite these similarities between HIn and HELP, HIn showed a significant difference in the thermal transition properties with respect to HELP. Turbidity measurements were performed to investigate the thermo‐responsive behavior of the biopolymers undergoing the inverse temperature transition in the absence and in the presence of near‐physiological salt concentration. It has to be taken into account that HELP, different from most of the other ELPs, possesses also cross‐linking domains, mimicking the tropoelastin structure. It is described that the optimal tropoelastin coacervation occurs in the presence of the near‐physiological salt concentration.^[^ ^18^ ^]^ We already reported that the presence of the cross‐linking domains in the HELP biopolymer affected its phase transition behavior and that the presence of a nearly physiological salt concentration fully restored the transition capacity.^[^ ^19^ ^]^

**Table 1:** Chemico‐physical parameters were obtained using Expasy Tools (ProtParam on‐line software) and the prediction of secondary structure using GOR IV was compared to the values obtained from CD measurements.

	p.I.	Hydropathy Index (GRAVY)	% Polar a.a.	% Charged a.a.		Α (%)	β (%)	Random coil (%)
**HIn**	12.2	0.97	2.5	4.4	GOR IV	27	5	68
					CD	30	6	74
**HELP**	11.7	1.10	1.9	3.2	GOR IV	26	4	70
					CD	29	10	61

Figure 3B shows the turbidity profiles of 2 mg mL^−1^ HIn in buffer solution with and without 0.15 M NaCl as a function of temperature. HIn showed no thermal transition up to 45 °C in the absence of salt, while in the presence of 0.15 M NaCl, a sharp increase in turbidity ≈31.8 °C was observed. In the presence of NaCl, HIn showed a sharper transition curve that occurred 10 °C earlier with respect to HELP (Figure 3B).

DSC peaks and onset temperatures of the inverse transitions are listed in **Table** **2** (see also Supporting Information) and were in agreement with those observed in the turbidity analysis. As already reported, the transitions of HELP and its modifications are always characterized by a broad peak extending over 10 °C or more^[^ ^19^, ^20^ ^]^ In the analysis presented here, especially in the presence of NaCl, the two biopolymers showed significant differences, confirming those observed in the turbidity experiments. Again, HIn in the absence of salt did not show any transition, even when heated at 90 °C (Table 2), confirming the results reported above by turbidimetric analyses. The possible explanation is based on the observation that the In domain contains several highly hydrophobic tryptophans and charged lysine and arginine. Being these amino acids endowed with diametrically opposed properties, the HIn hydropathy index resulted diminished respect to HELP (Table 1). In general, the T_t_ of the ELPs is lowered when the hydrophobicity of the chain is raised, for example, by substitution of the fourth, “guest” residue of their pentapeptidic motif.^[^ ^21^ ^]^ On the other hand, an analogous but distinct effect was also observed, consisting of the decrease of the ELP T_t_ by the fusion with hydrophobic moieties.^[^ ^22^ ^]^ Consistent with these observations, the increased sharpness of the HIn transition profile as well as the decreased transition temperature suggested that HIn responded more promptly than HELP to temperature rise, likely due to the hydrophobicity increase of the macromolecule. However, due to the presence of the cross‐linking regions in HELP domain, this behavior is evidenced only when the charges are shielded avoiding repulsion, such as in the presence of salt.

**Table 2:** DSC onset and peak inverse transition temperatures for the 8 mg mL^−1^ solutions of HIn and HELP biopolymers in the absence and in the presence of 0.15 M NaCl.

	*T* [°C]	NaPi buffer	NaPi buffer / 0.15 M NaCl
**HIn**	onset	–	19.4
	peak	–	29.8
**HELP**	onset	16.2	31.4
	peak	29.5	35.6

In summary, the HIn construct carrying the antimicrobial domain placed at the C‐terminus of HELP was successfully produced by recombinant methods. This construct was fluorescent, due to the presence of the tryptophan‐rich In domain. This convenient feature allowed the tracking of the domain after its release from the HIn biopolymer. The phase transition behavior conferred by the HELP domain was maintained and even enhanced in the fusion construct, as demonstrated by the physicochemical characterization. Consequently, the purification of the construct by exploiting this property resulted in an average yield on the lab scale of more than 200 mg per liter of bacterial culture.

### 2.2 Antimicrobial Activity of HIn

Around 10 ELP fusion constructs carrying the AMP at the N‐terminus have been described to possess antimicrobial activity without the need to leave the antimicrobial domain (^[^ ^10^ ^]^ and references herein). By contrast, only one ELP fusion carrying the AMP at the C‐terminal was described to be active.^[^ ^23^ ^]^ In our system, the In domain is placed at the C‐terminal part of the fusion protein exploiting the features of the HELP vector (see Supporting Information). Thus, we assayed the whole construct for the activity against bacterial models that are commonly used as reference strains for antibiotic susceptibility testing.

**Figure 4:**
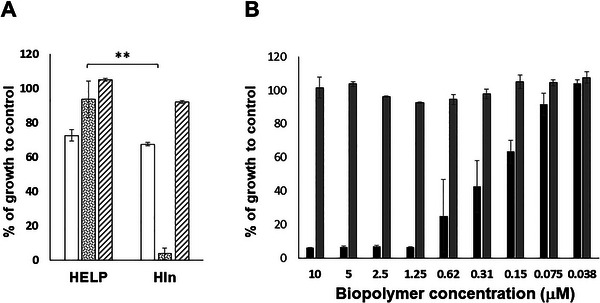
Antimicrobial activity of the HIn biopolymer. A) Comparison of the effect on bacterial growth of 20 µM of HELP and HIn biopolymers toward three bacterial strains. White, *S. aureus*; dotted, *P. aeruginosa* and hatched, *E. coli*. B) Antimicrobial activity evaluation of the HIn (black) fusion protein compared to the HELP (grey) biopolymer toward *P. aeruginosa*. The values were expressed as the percent respect the growth of the untreated control and represented the mean ± SD, *n* = 4; **p ≤ 0.001.

The activity of the purified HIn biopolymer, as well as that of the HELP alone, was assayed toward *S. aureus*, *P. aeruginosa*, and *E. coli*. The results are presented in **Figure** **4**A. At the concentration of 20 µM, HELP did not affect the growth of the tested microorganisms. On the contrary, efficient antimicrobial activity of the HIn biopolymer was observed at this concentration against *P. aeruginosa* (Figure 4A). One possible explanation is that *P. aeruginosa*, under certain growth conditions, can secrete elastolytic enzymes,^[^ ^24^ ^]^ that may trigger In release in the medium, thus hindering the microorganism growth. This observation prompted us to select this microorganism as the model for this study, and we performed the mInimum inhibitory concentration (MIC) assay with the HIn biopolymer (Figure 4B). No significant effect was observed for any concentration of HELP relative to the untreated control, whereas HIn displayed the inhibitory effect on this strain at the concentration of 1.25 µM.

**Figure 5:**
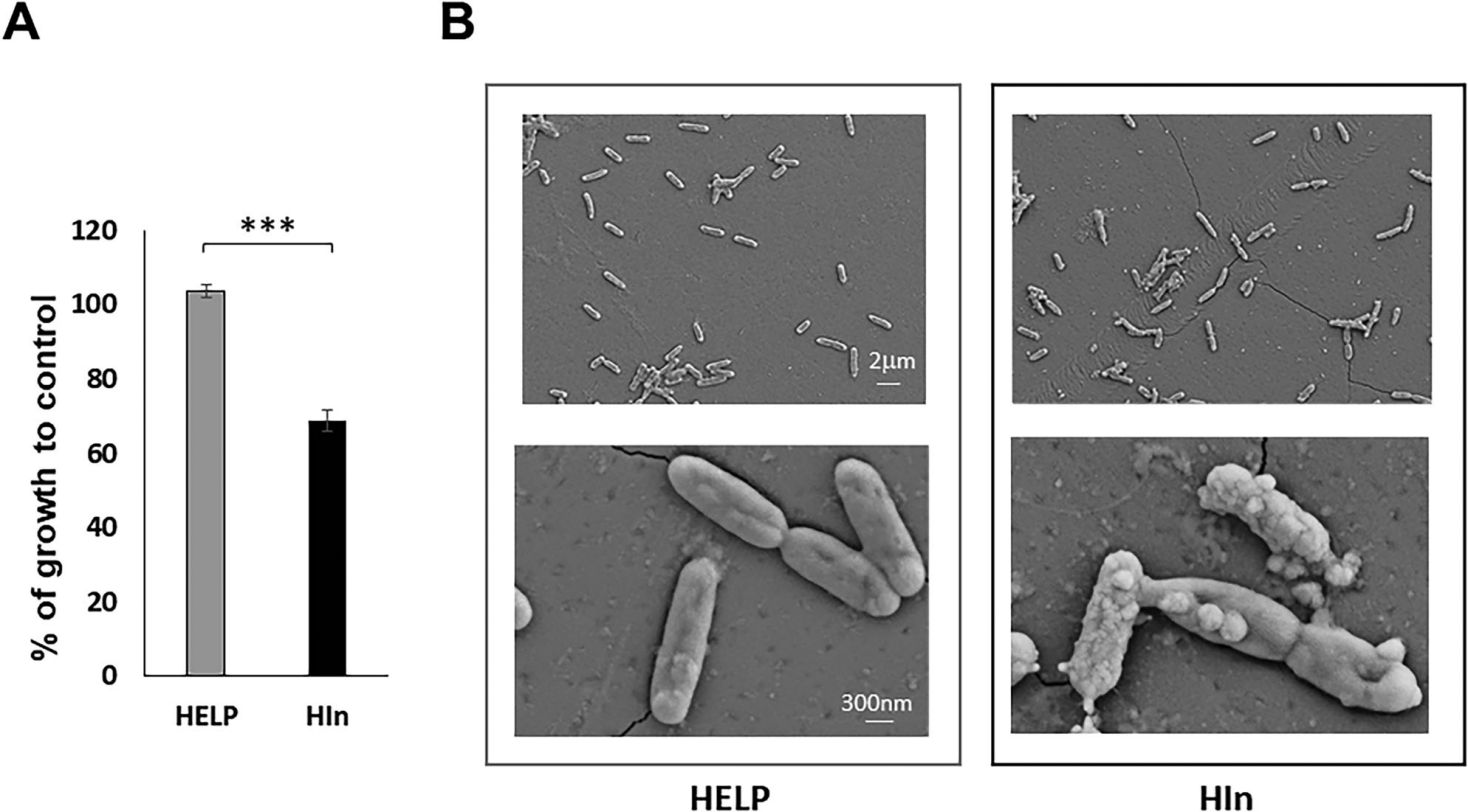
Antimicrobial capacity of HIn‐based coatings. A) Effect of HELP and HIn coatings *on P. aeruginosa* growth. Results were expressed as the percent respect the growth of the control on the uncoated surface and represented the mean ± standard deviation (SD) of at least three replicates. ***p ≤ 0.0001. B) Representative images of the SEM analysis of *P. aeruginosa* deposited on the HELP and HIn‐based coatings.

After the finding that HIn biopolymer maintained the activity conferred by the fusion AMP, we explored the opportunity to prepare antimicrobial surfaces. To coat the surface, aqueous solutions of HIn and HELP were deposited and dried on the bottom of 24‐well plates. *P. aeruginosa* cells re‐suspended in NaPi buffer (detailed in the Materials and Methods section) were deposited on each coating or on the untreated control surface. HELP‐coated surfaces did not affect bacterial growth compared to the uncoated surfaces, whereas HIn‐coated surfaces showed a bacterial growth reduction of ≈30%, suggesting a bactericidal activity due to the presence of the In domain (**Figure** **5**A). SEM analysis was performed on these samples to observe any effect of the contact between the bacteria and the coated surface (Figure 5B). The bacteria deposited and incubated on HELP coating showed the morphology expected for healthy growing cells (Figure 5B left panel) whereas most of the bacteria deposited on the HIn‐based coating showed extended membrane blebbing, indicating that cells underwent disruption (Figure 5B right panel). These results clearly showed that In, although in fusion with HELP, was able to display its effect at least toward *P. aeruginosa*, opening the possibility to employ HIn to realize materials and surfaces endowed with antimicrobial activity.

### 2.3 Specific Release of the In Domain from the HIn Biopolymer

**Figure 6:**
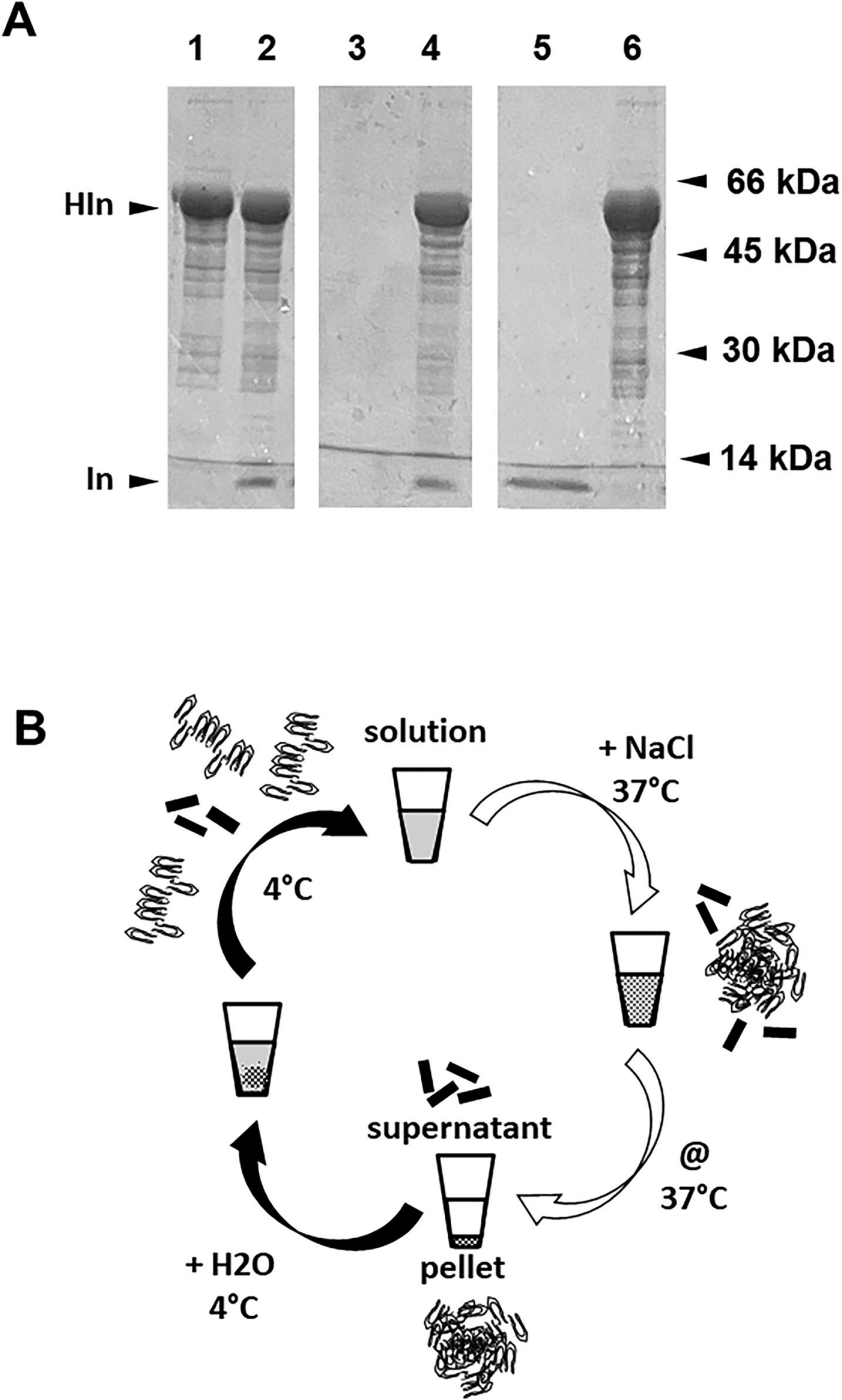
Release of In domain by specific enzymatic cleavage of HIn. Representative image of SDS‐PAGE analysis of the HIn biopolymer treated with the Glu‐C endoprotease. Lane 1, untreated HIn biopolymer; lane 2, HIn biopolymer treated with Glu‐C; lane 3, supernatant after the precipitation and centrifugation at 37 °C; lane 4, pellet after the precipitation and centrifugation at 37 °C; lane 5, supernatant after the precipitation and centrifugation at 37 °C in the presence of detergent; lane 6, pellet after the precipitation and centrifugation at 37 °C in the presence of detergent. B. Scheme of the purification process based on the ITC method of the HELP biopolymer, which is soluble in a cold solution and precipitates after temperature and ionic strength increase. Molecular masses of the protein markers are indicated on the right. Bovine serum albumin (66 kDa), ovalbumin (45 kDa), carbonic anhydrase (30 kDa), lysozyme (14 kDa). Coomassie blue staining.

Our model for AMP production in fusion with an ELP carrier was designed to allow the specific release of the bioactive domain using a specific endoprotease, the Glu‐C enzyme that cleaves the proteins after the glutamic acid (Figure 1). Since HELP does not contain any glutamic or aspartic acid, no cleavage of the HELP domain is expected after the treatment with this enzyme, and this was confirmed by the SDS‐PAGE analysis (not shown). When the reaction was performed in solution, the release of In could not be followed by fluorescence since HIn shares the same fluorescence property of In. For this reason, SDS‐PAGE analysis was performed to assess the release of In. After Glu‐C reaction, an electrophoretic band migrating faster than the front was clearly visible, suggesting the release of the In domain (**Figure** **6**A, lane 2).

At first, the thermo‐responsive behavior of HELP was employed for the Inverse Transition Cycling (ITC) purification of the released In domain (Figure 6B). However, after the addition of NaCl and warming to 37 °C to elicit the phase transition of HELP, and with subsequent centrifugation, no electrophoretic signal was detected in the supernatant (Figure 6A, lane 3) whereas both HELP and In were found in the pellet (Figure 6A, lane 4). This indicated that, in these conditions, the highly hydrophobic, tryptophan‐rich, poorly soluble In domain remained in the pellet, likely due to hydrophobic interaction with the HELP moiety. In fact, when the phase transition was induced in the presence of detergent, after the centrifugation, the band corresponding to the In domain was clearly visible in the supernatant (Figure 6A, lane 5) and it was totally absent in the pellet constituted by HELP alone (Figure 6A, lane 6). Quantification of In in the supernatant with the BCA assay resulted in an average of 35 µg of In per mg of HIn, very close to the theoretical yield value, keeping into account that In represents ≈1/27 of the whole macromolecule. However, due to the presence of the detergent, it was not possible to assess the antimicrobial activity.

### 2.4 HIn Hydrogel Matrix Production and Bioactivity

**Figure 7:**
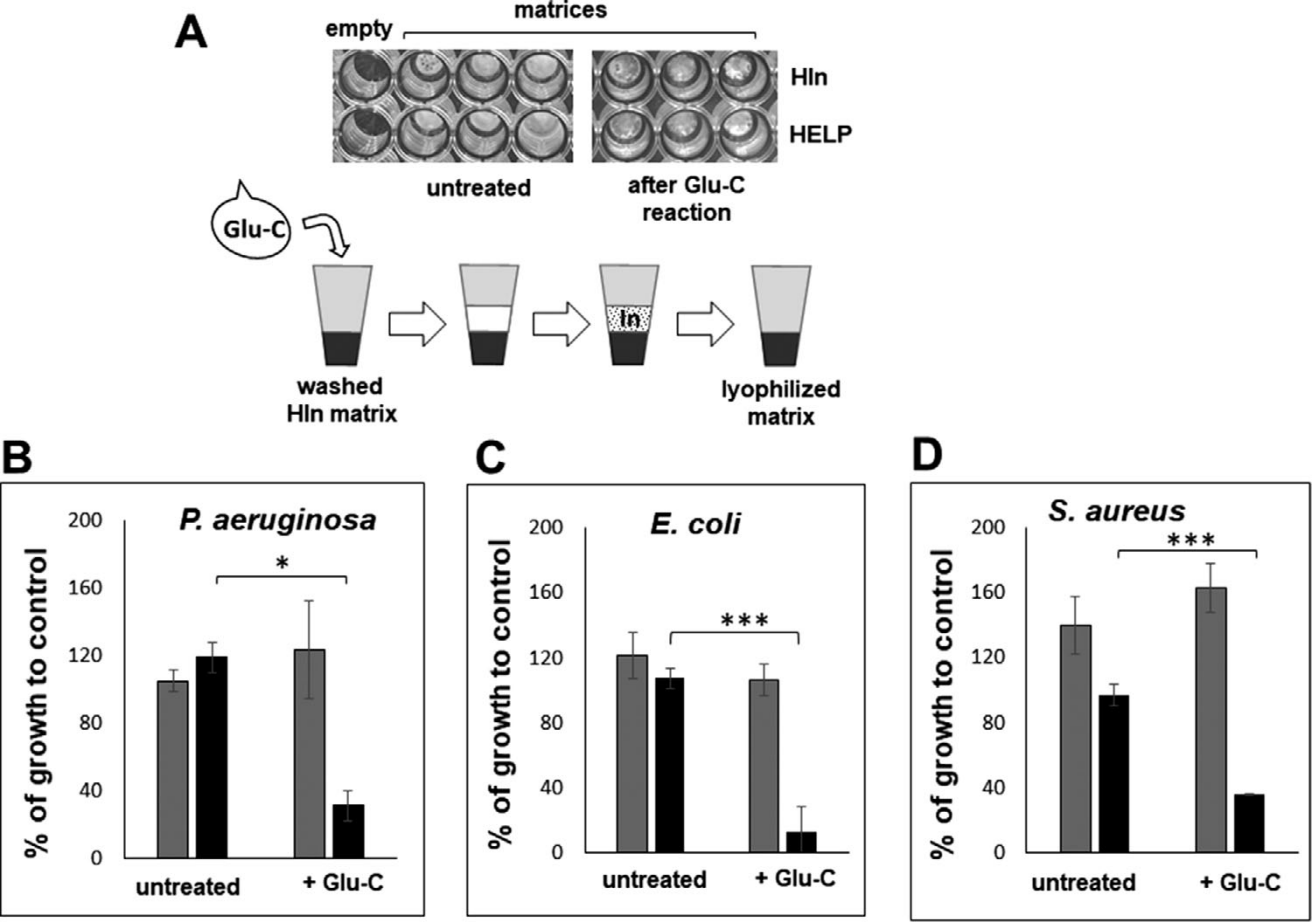
Antimicrobial activity of the HIn matrix. A) Macroscopic aspect of the HIn and HELP matrices before and after Glu‐C reaction (top) and schematization of matrix treatment to test the activity toward the bacteria (bottom). B) Antimicrobial activity of the HELP (grey) and HIn (black) matrix toward *P. aeruginosa*. C. Antimicrobial activity of the HELP (grey) and HIn (black) matrix toward *E. coli*. D. Antimicrobial activity of the HELP (grey) and HIn (black) matrix toward *S. aureus*. The values were expressed as the percent respect the growth of the untreated control and represented the mean ± SD, *n* = 4; *p ≤ 0.01, ***p ≤ 0.0001.

The matrix‐forming property of HELP has already been described. The method is based on the covalent binding of the biopolymer chains.^[^ ^13^ ^]^ Since the HELP cross‐linking domains are the main target of the transglutaminase enzyme that forms the matrix network, it is likely that any domain in fusion with HELP will not significantly affect the reaction, since most probably it is not the preferred substrate of the enzyme, even if it contains a few lysines and glutamines. Thus, the enzymatic cross‐linking of a solution of 4% of HIn was performed, showing the same macroscopic aspect of the 4% HELP matrices that were prepared in parallel as the control (**Figure** **7**A, see Materials and Methods for details).

The use of the specific endoprotease Glu‐C was expected to trigger the release of the In domain from the HIn matrix. Following the cross‐linking and extensive washing, and after assessing the absence of any unreacted substrate by fluorescence, the HIn matrix was treated with Glu‐C. After the proteolytic reaction, all the matrices were macroscopically intact, indicating that the cleavage was specific, as expected (Figure 7A). The supernatant was collected and analyzed by fluorescence, SDS‐PAGE, and mass spectrometry. Comparing the wash of the matrix before Glu‐C addition with the supernatant after the proteolytic reaction, a fluorescence signal was detected in the latter, suggesting the presence of the In domain in the solution (not shown). The ESI‐MS confirmed the presence of one predominant component of 1907.02 Da in the supernatant, corresponding to the expected value of 1907.30 Da calculated based on the amino acid sequence of In (Figure S3, Supporting Information). The supernatant was then tested for antimicrobial activity against *P. aeruginosa*, and a MIC value of 3 µM was determined, corresponding to the same order of magnitude of the data reported in the literature for In.^[^ ^25^ ^]^

The antibacterial capacity of HIn matrices was assayed. After the cross‐linking reaction, both 4% HELP and HIn matrices were extensively washed with water to remove any unreacted substrate and then they were lyophilized (Figure 7A). The matrices were re‐hydrated in bacterial culture, maintaining the same conditions of the MIC assay (detailed in the Materials and Methods section). Despite the fact that the uncleaved HIn biopolymer displayed antimicrobial activity toward *P. aeruginosa* (Figure 4), both HELP and HIn matrices allowed bacterial growth, suggesting that tethering of the In domain in a matrix may inactivate it. This was described for cationic peptides, since it was observed that peptide immobilization on a surface could result in a pronounced activity decrease.^[^ ^26^ ^]^ Thus, the cleavage with Glu‐C was performed on the HIn as well as the HELP matrix. All the samples were lyophilized, to be re‐hydrated in the presence of *P. aeruginosa*, as described above for the undigested matrices. The results are shown in Figure 7B. The untreated and the Glu‐C treated HELP matrices as well as the untreated HIn matrix did not show any significant effect on bacterial growth. On the contrary, the HIn matrix treated with Glu‐C drastically reduced the growth of *P. aeruginosa*, suggesting the release of the active antimicrobial domain from the HIn matrix. Since it is described that In has a broad activity toward bacteria, we repeated the same assay with *S. aureus* and with *E. coli*. The results in Figures 7C,D clearly showed that the In activity toward these two strains was restored after the cleavage with Glu‐C of the HIn matrix.

Overall, the fusion of In with HELP has demonstrated the versatility of this peculiar elastin‐like domain as a valuable carrier. First, the cleavage of In from the matrix and its release was successfully performed representing a convenient and smart alternative option with respect to ITC to obtain the bioactive AMP domain. Second, although the matrix resulted not active after the cross‐linking, we demonstrated that the antimicrobial property was restored after the specific proteolytic cleavage. Since In to exert its effect has to penetrate inside the bacterial cell, the antimicrobial activity was just hindered when the AMP resulted immobilized within the matrix.

This is consistent also with the observation that the HIn coating was toxic for bacterial cells. In the coating HIn is not cross‐linked, thus it is likely that it could re‐dissolve in aqueous solution to some extent and, in consequence, it can be internalized by the bacteria exerting the observed growth inhibition (Figure 5).

The features of the HELP fusion with an AMP domain can be exploited to realize smart stimuli‐responsive materials with antimicrobial properties that can be triggered on/off on demand.

### 2.5 Release of the Antimicrobial Activity by Elastase

An interesting feature of HELP biopolymer is its elastin‐mimicking structure, which is composed of both elastin‐like and cross‐linking domains. It has already been reported that HELP is particularly susceptible to elastase activity, likely due to the presence of the cross‐linking domains that represent one of the main targets of this enzyme.^[^ ^27^ ^]^

**Figure 8:**
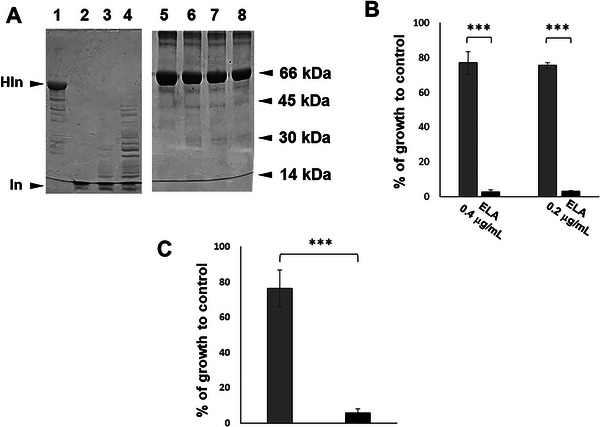
HIn biopolymer and HIn matrix degradation by elastase and release of the antimicrobial activity. A) Representative SDS‐PAGE analysis of elastolytic degradation of the HIn biopolymer. HIn (lane 1) was treated with 0.4, ng µL^−1^ of elastase (lane 2), 0.2, ng µL^−1^ of elastase (lane 3), and 0.1, ng µL^−1^ of elastase (lane 4). In the same conditions, BSA (lane 5) was treated with equal amounts of elastase, 0.4, ng µL^−1^ of elastase (lane 6), 0.2, ng µL^−1^ of elastase (lane 7) and 0.1, ng µL^−1^ of elastase (lane 8). Molecular masses of the protein markers expressed in kilodaltons (kDa) are indicated on the right, Coomassie blue staining. B. Effect on bacterial growth of HELP (grey) and HIn (black) biopolymer solution treated with 0.4 and 0.2 ng µL^−1^ of elastase (ELA). C. Effect of HELP (grey) and HIn (black) matrix degradation by elastase on bacterial growth. The values were expressed as the percent respect the growth of the untreated control and represented the mean ± SD, *n* = 4; ***p ≤ 0.0001.

This property is valuable to realize stimuli‐responsive systems for the sustained release of active AMPs, especially in the presence of elastolytic activity, a condition occurring during neutrophil activation due to inflammation processes, for instance. Therefore, we assessed the release of In by elastase by first testing the HIn biopolymer in solution (**Figure** **8**A). Elastase concentrations of 0.4 and 0.2 µg mL^−1^ were necessary to degrade all the HELP domain of the HIn biopolymer, releasing the In product (Figure 8A, lanes 2 and 3), whereas the same enzyme concentrations did not degrade bovine serum albumin (BSA) at all (Figure 8A, compare lane 5 with lanes 6–8), confirming the reaction specificity.

The antimicrobial activity of these reactions’ products was assessed against *P. aeruginosa* and the data showed that the presence of the products of the elastolytic degradation of HIn biopolymer resulted in a marked inhibition of bacterial growth. In contrast, the degradation products of HELP had only a minor effect (Figure 8B).

Moreover, the 4% matrix of HIn and that of HELP, were treated with elastase. The reaction completely degraded the matrices, as already reported.^[^ ^27^ ^]^ The obtained supernatants were then assayed with *P. aeruginosa*, showing that HIn matrix degradation was sufficient to allow the release of an amount of In that was effective in inhibiting bacterial growth (Figure 8C).

These data showed that our HIn construct might be employed to obtain smart materials that, upon the proper stimuli, trigger the release of a bioactive compound that may interfere with the process that provoked the release, possibly leading to the attenuation of the process itself. The concentration of the enzyme that showed to be sufficient to release an amount of bioactive domain, was ineffective on the BSA, suggesting that the high susceptibility of HELP to elastase is a valuable feature to exploit for the production of antimicrobial materials that may be well tolerated by the tissues.

## 3 Conclusion

The HELP fusion platform described in this work was successfully employed to produce a model construct bearing the bioactive antimicrobial domain indolicidin. The synthetic gene was further tailored to introduce a specific cleavage site, giving an example of the versatility of our system. The HIn recombinant biopolymer retained both the properties of the elastin‐like carrier, as demonstrated by the physicochemical characterization, and those of the antimicrobial domain, displaying growth inhibitory activity at least towards one of the model strains tested. The exploitation of the HIn thermo‐responsive behavior allowed its production with very good purity and yield on a lab scale and can be further scaled up. The matrix‐forming property of HELP was also maintained by HIn. Two ways were evaluated to produce indolicidin by a specific enzymatic cleavage route. To purify the In released from the reaction the ITC was exploited to deplete the supernatant of the HELP moiety. Although this method showed some limitations due to the high hydrophophobicity and poor water solubility of In itself, the bioactive AMP was successfully recovered by cleaving the HIn matrix and recovering the supernatant. Overall, the recombinant platform described herein showed valuable versatility and opened the door to the production of materials endowed with antimicrobial properties that might be integrated into engineered multifunctional bio‐systems capable of sensing and actively responding to their surrounding environment. Due to the strict control of the amino acid sequences, this platform can also be exploited to better elucidate the still poorly characterized mechanism of action of the AMPs at the molecular level.

## 4 Experimental Section

### Antimicrobial HELP Cloning, Expression, and Purification

The sequence coding for indolicidin (In, GenBank: AAB21494.1) was in‐frame cloned at the C‐terminal region of HELP gene exploiting the unique *DraIII – HindIII* restriction sites.^[^ ^12^ ^]^ The positive clones were sequenced to verify the open reading frame and then the *E. coli* C3037I (New England Biolabs Inc., Ipswich, MA) expression strain was transformed with the plasmid carrying the new HIn construct. The expression and purification of the recombinant HELP and HIn biopolymer were performed in standard conditions, as described previously.^[^ ^28^ ^]^

The purified biopolymers were analyzed by a 9% acrylamide SDS‐PAGE stained by Coomassie blue using a reported procedure.^[^ ^19^ ^]^ The purified products were lyophilized for long‐term storage.

### Physicochemical Characterization

The spectrophotometric analysis of 2 mg mL^−1^ aqueous solution of the pure samples was performed in the range of λ = 250 – 300 nm using a Jenway scanning spectrophotometer (Model 7205, Jenway, Staffordshire UK). The fluorescence of aqueous solutions of the biopolymers at different concentrations was measured in a 96‐well black polystyrene microplate (Nunc, Sjelland, Denmark), analyzing 50 µL well^−1^ and detecting the emission fluorescence at λ = 350 nm, following excitation at λ = 280 nm by a microplate reader (Synergy H1, BioTek, Winooski, VT).

The secondary structure was evaluated using the ProtParam (Expasy) program available on the SIB Swiss Institute of Bioinformatics website. The grand average of hydropathy value (GRAVY) for proteins was calculated as the average of hydropathy values of all the amino acids in the sequence. The prediction of secondary structures of HIn was based on the primary amino acid sequences of the polypeptides by using GOR IV software from the Expasy Web site (http://www.au.expasy).

Circular dichroism (CD) spectra were recorded on protein solutions with a concentration of 0.1 mg mL^−1^ in NaPi (10 mM sodium phosphate pH = 6.8) or NaPi / NaCl (10 mM sodium phosphate/ 0.15 M NaCl pH = 6.8) buffers. The CD Spectra were recorded at 25 °C in a 200‐ to 500‐nm thermostatic cell on a Jasco J‐710 spectrometer under constant nitrogen flow, and the data were expressed as the mean molar ellipticity [θ] of the residue (mdeg‐cm^2^‐dmol^−1^).

By using a turbidimetric method, the optical density (OD) of HIn samples was measured at *λ* = 350 nm in the range of temperature 20−50 °C at a heating scan rate of 0.2 °C min^−1^ on a Jenway 6300 spectrophotometer. The inverse transition temperature (*T*_t_) was obtained as the temperature corresponding to 50% of the maximum OD value. The purified proteins were dissolved in NaPi buffer to a final concentration of 2 mg mL^−1^. The solutions were equilibrated at 4 °C for 16 h before analysis.

The thermal properties of lyophilized proteins in solution were evaluated by Differential Scanning Calorimetry (DSC) using a Setaram MicroDSC III DSC model. Stainless steel cells were filled by weight with protein samples (4 mg mL^−1^, in NaPi or NaPi /NaCl buffers) and then hermetically sealed and equilibrated for 16 h at 4 °C. The calorimeter was pre‐equilibrated at 5°C for 10 min, followed by heating from 5 to 70 °C at a scan rate of 0.5 °C min^−1^. The solvent was used as a reference. The calorimetric area, the onset, and the peak inverse transition temperatures (*T*_t_) were determined by a homemade software written in Fortran code.

### Assessment of Antimicrobial Activity of the HIn Biopolymer and of the Derived Materials

The bacterial strains used to assess the antimicrobial activity of the HIn recombinant biopolymer were *S. aureus* ATCC 25 923, *P. aeruginosa* ATCC 15 692, and *E. coli* ATCC 25 922. The antimicrobial activity was assessed using a modification of the broth microdilution method, according to Bera et al.^[^ ^29^ ^]^ using 2.1% (w/v) Mueller‐Hinton broth (Merck Millipore, Massachusetts, USA). The bacterial cells were incubated with the biopolymers in the NaPi buffer (10 mM sodium phosphate, pH 6.8) before broth addition and incubation at 37 ˚C. The minimum inhibitory concentration (MIC) was taken as the lowest concentration of the biopolymer that completely inhibited the growth of microorganisms was assessed as follows. Briefly, 300 µL of overnight bacterial culture were diluted in 10 mL of 2.1% (w/v) Mueller‐Hinton broth and incubated at 37 ˚C with continuous shaking (150 rpm) for ≈2–2.5 h, until OD ≈ 0.5 was reached. At this point, 50 µL of the bacterial culture were diluted in 10 mL of NaPi buffer, corresponding to ≈2.5 × 10^6^ CFU mL^−1^ (bacterial working solution). Lyophilized biopolymers, previously sterilized by 0.22 µm filtration, were re‐dissolved to a concentration of 100 µM in sterile NaPi to prepare two‐fold serial dilutions that were deposited (10 µL per well) in a sterile 96‐well polystyrene U‐shaped bottom wells microplate (Sarstedt, Numbrecht, Germany).

Right after, 20 µL of bacterial working solution were added per well and incubated for 1 hour at room temperature (21–23 °C). Sterile NaPi buffer was used as the control. Then, 70 µL of 3% (w/v) Mueller–Hinton broth were added to each well to a final volume of 100 µL of 2.1% (w/v) Mueller‐Hinton broth (final concentration). The final concentration of the biopolymers ranged from 10 to 0.038 µM and the final seeding density was 5 × 10^5^ CFU mL^−1^. The microwell plates were incubated at 37 °C for 20 hours, and then they were analyzed at 600 nm in a microplate.

### Antimicrobial Activity of HIn Coatings

Coatings were prepared either in the bottom of the well of 24‐well tissue culture‐treated polystyrene microplates (Sarstedt, Numbrecht, Germany) for assessment of antimicrobial activity, or in 13 mm‐diameter sterile plastic coverslips (# 83.1840.002, Sarstedt, Numbrecht, Germany) for SEM analysis. Lyophilized HELP and HIn biopolymers were re‐dissolved in water and sterilized by filtration (0.22 µm). Coatings were prepared in the form of thin films by deposition of 50 µL of 2 mg mL^−1^ biopolymer sterile solution, thus covering a surface of 1 cm^2^ with 100 µg of biopolymer. Samples were dried allowing water to evaporate at room temperature under a sterile hood.

To evaluate the antimicrobial capacity of the biopolymer coatings, a modification of the antimicrobial assay described above was set up. Briefly, 20 µL of 10 mM NaPi buffer containing approximately 2,5 × 10^6^ CFU mL^−1^ of *P. aeruginosa* ATCC 15 692 strain were deposited within the coated surfaces of the 24‐well polystyrene microplate and were incubated for 1 hour at room temperature (21–23 °C). Right after, 300 µL of 2.1% (w/v) Mueller‐Hinton broth were added to the wells and the microplate was incubated at 37 ˚C for 20 h. The bacterial growth was determined by reading 50 µL of each sample in a U‐shaped 96‐well polystyrene microplate at 600 nm in a microplate reader.

For SEM analysis, *P. aeruginosa* ATCC 15 692 culture was diluted to 2 × 10^8^ CFU mL^−1^ in NaPi buffer, and 20 µL were deposited onto the coated surface of the plastic coverslips and incubated for 1 hour at room temperature. After incubation, the solution was removed by washing twice with 50 µl PBS. The samples were fixed at room temperature for 30 min using 50 µL of 2.5% (v/v) glutaraldehyde in PBS. After glutaraldehyde removal, the coverslips were rinsed again by soaking in PBS. Samples dehydration was performed with increasing concentrations of ethanol/water (30%, 50%, 70%, 90%, and 100%) using for each Step 1 mL of solution and 5 min of incubation. Subsequently, samples were dried under the hood flow, mounted on aluminum stubs covered with a double‐sided carbon tape and coated with chromium using a Q150T ES plus sputter coater (Quorum Technologies Ltd., UK). The morphological analysis was then performed with a scanning electron microscope (Gemini 300, Zeiss, Germany) working in secondary electron detection mode. The working distance was set at about 8.5 mm to obtain the appropriate magnifications, and the acceleration voltage was set at 5 kV.

### Antimicrobial Capacity Assessment of the HIn Matrix

To assess the antimicrobial activity of the HIn matrix, conditions similar to those described above for the MIC assay were adopted.

HELP and HIn hydrogel matrices were prepared in a sterile 96‐wells polystyrene V‐shaped bottom microplate (Sarstedt, Numbrecht, Germany). 10 µL of 4% (w/v) sterile aqueous solutions of HELP and HIn were deposited in the well bottom and were enzymatically cross‐linked using microbial transglutaminase (N‐Zyme Biotec GmbH, Darmstadt, Germany) at a final concentration of 2 µg µL^−1^. Cross‐linking was carried out for 1 hour at room temperature (21–23 °C) and after three washes with excess of water, the matrices were directly lyophilized or treated with Glu‐C. For this latter reaction 25 µL of 100 mM ammonium bicarbonate pH 8 with 10 ng µL^−1^ Glu‐C were added to each matrix, whereas the control samples were treated with 25 µL of buffer alone and incubated overnight at 37 °C. After this reaction, the microplate was transferred at −80 °C and lyophilized.

Subsequently, each matrix was re‐hydrated with 20 µL of bacterial working solution (2.5 × 10^6^ CFU mL^−1^ in 10 mM NaPi buffer) and incubated for 1 h at room temperature before the addition of 100 µL of 2.1% (w/v) Mueller‐Hinton broth. The microplate was incubated for 20 h at 37 °C and then the supernatants were transferred in a U‐shape microplate to be read at 600 nm.

### Specific Release of the In Antimicrobial Domain by Glu‐C Endoprotease from the HIn Biopolymer

The reactions with the Glu‐C enzyme (New England Biolabs, #P8100S) were set up in parallel with the HELP and the HIn biopolymers at the final concentration of 6 mg mL^−1^ in 100 mM ammonium bicarbonate buffer, pH 8, with of Glu‐C 10 ng µL^−1^. After the incubation at 37 °C for 5 h, 1.5 µL of the reaction were added to the same volume of Laemmli loading buffer for the SDS‐PAGE analysis.

To separate the In peptide from the HELP moiety, a purification exploiting the inverse transition cycling was performed, adding to the reaction NaCl to 1.5 M, Triton‐X100 to 1% (v/v), and warming at 37 °C for 5 min. After the centrifugation for 5 min at 10 000 rpm. The supernatant and the pellet were separated and subjected to SDS‐PAGE analysis.

### Specific Release of the In Domain by Glu‐C Endoprotease from the HIn Matrix

The In domain was released from the 4% (w/v) HIn matrix by incubation with Glu‐C. Matrices of 150 µL each were incubated overnight at 37 ˚C with 500 µL of 100 mM ammonium bicarbonate buffer pH 8 with 10 ng µL^−1^ of Glu‐C enzyme. The control samples were incubated in the buffer alone.

After the overnight incubation, the supernatant was collected. Then, each matrix was washed twice for 1 h with 1 mL of 20% (v/v) acetonitrile in water. A third wash was performed overnight. The washes that showed fluorescence emission were pooled together with the supernatant to be frozen and lyophilized. The lyophilized material was re‐suspended in water to be assayed by Bicinchoninic acid assay (Pierce BCA Protein Assay Kit), SDS‐PAGE, and ESI mass spectrometry. For the antimicrobial activity assessment, the lyophilized material was re‐suspended in NaPi and sterilized by 0.2 µm filtration.

### Release of the In Domain from the HIn Biopolymer by Elastolytic Activity

The HIn biopolymer was sterilized by filtration and a 100 µM solution in NaPi buffer was treated with 0.4, 0.2, and 0.1 ng µL^−1^ of elastase (Sigma, elastase from porcine pancreas, #E7885) for 3 h at 37 °C in a final reaction volume of 30 µL. In parallel, the same conditions were adopted to set up equivalent reactions with the bovine serum albumin (BSA) as well as the HELP biopolymer. A 2 µL of each reaction were analyzed in SDS‐PAGE. The antimicrobial capacity of 10 µL of the elastase reaction mixtures were assessed towards *P. aeruginosa* ATCC 15 692 as described above.

### Release of the In Domain from the HIn Matrix by Elastolytic Activity

4% (w/v) HIn hydrogel matrices (10 µL each) were prepared in sterile conditions as described above. After the cross‐linking, the matrices were extensively washed with sterile NaPi buffer and then incubated with 15 µL of 8 ng µL^−1^ elastase solution in NaPi buffer at 37 °C for 3 h. These reactions were then incubated with 10 µl of *P. aeruginosa* ATCC 15 692 bacterial solution diluted to 5 × 10^6^ CFU mL^−1^ in NaPi buffer for 1 hour at room temperature before the addition of 70 µL of 3% (w/v) Mueller–Hinton broth. The microwell plates were incubated at 37 °C for 20 h, and then they were analyzed at 600 nm in the microplate reader.

### Statistical Analysis

One‐way analysis of variance (ANOVA) was carried out to compare the means of different data sets within each experiment. A value of *p* < 0.05 was considered statistically significant. All experiments were performed at least in triplicate.

## Conflict of Interest

The authors declare no conflict of interest.

## Supporting information

Supporting Information

## Data Availability

The data that support the findings of this study are available from the corresponding author upon reasonable request.
