# Supplementary material for: A Versatile Elastin‐Like Carrier for Bioactive Antimicrobial Peptide Production and Delivery
Source: Macromol Biosci. 2023 Sep 24;24(3):2300236. doi: 10.1002/mabi.202300236 (PMC13420748; doi:10.1002/mabi.202300236)
Supplement: Supplementary file 1 — Supporting Information [file MABI-24-2300236-s001.pdf]

Macromolecular Bioscience

Supporting Information

for *Macromol. Biosci.*, DOI 10.1002/mabi.202300236

A Versatile Elastin-Like Carrier for Bioactive Antimicrobial Peptide Production and Delivery

*Laura Colomina – Alfaro, Paola Sist, Silvia Marchesan, Ranieri Urbani, Artemis Stamboulis and Antonella Bandiera**

A versatile elastin-like carrier for bioactive antimicrobial peptide production and delivery

Laura Colomina – Alfaro, Paola Sist, Silvia Marchesan, Ranieri Urbani, Artemis Stamboulis, and Antonella Bandiera

Supporting Information

HIn primary structure

Domain	Theoretical MW	aa sequence
His-tag	1629.74 Da	MRGSHHHHHHGSAAA
HELP	43117.88 Da	$(AAAAAAKAAAKAAQFGLVPGVGVAPGVGVAPGVGVAPGVGLAPGVGVAPGVGVAPGVGVAPGIAP)_8$
Linker	2558.92 Da	GGLAAAAAAAAAKAAAKAAQGGLPGIPGRE
AMP (In)	1907.30 Da	ILPWKWPWWPWRR

Cloning of HIn

To obtain the HIn construct, the coding sequence of the linker and of the indolicidin, flanked by *DraIII* sites was purchased from Eurofins Genomics and ligated into the pEX8EL vector exploiting the unique *DraIII* site at the HELP orf C-terminus [described in *Bandiera, A., et al., (2005) Expression and characterisation of human elastin repeat-based temperature responsive protein polymers for biotechnological purposes. Biotechnol. Appl. Biochem., 42, 247-256.*]. After transformation of *Escherichia coli C3037I* (New England Biolabs), positive clones were selected and verified by sequencing (Eurofins Genomics). The expression strain was transformed with the plasmid carrying the new HIn construct.

Expression of HIn

Selected clones of *E. coli* C3037I strain transformed with the plasmid carrying the new HIn construct were grown in Luria Bertani medium (LB, 10 g/L tryptone, 5 g/L sodium chloride and 5 g/L yeast extract, pH 7.2) supplemented with 50 µg/mL of ampicillin and 70 µg/mL of chloramphenicol. Typically, a starter culture of 120 mL of the same medium after overnight growth at 37°C was used to inoculate 1.2 L of Terrific Broth (TB, 12 g/L tryptone and 24 g/L yeast extract) supplemented with phosphate-buffered glycerol (PGB, 2.3 g/L potassium phosphate monobasic, 12.5 g/L potassium phosphate dibasic, and 4 mL/L glycerol). Bacterial cells were grown at 37 °C under shaking conditions until turbidity at 600 nm reached about 1 O.D. unit. The culture was then induced with Isopropyl β- d-1-thiogalactopyranoside to a final concentration of 0.1 mM and allowed to further grow for 5 h. Then, bacterial cells were collected by centrifugation at 8000 rpm for 20 min at 10°C (Beckman–Coulter, J-26 XP). The pellets were stored at -20°C for further processing.

Extraction and ITC purification of HIn product

The protocol exploited the thermo-responsive behavior of HIn and was based on the Inverse Transition Cycling (ITC) first described by *Meyer, D., Chilkoti, A. (1999). Purification of recombinant proteins by fusion with thermally-responsive polypeptides. Nat Biotechnol 17, 1112–1115 doi.org/10.1038/15100.*

The pellet obtained from 1.2 L of IPTG-induced bacterial culture was re-suspended in 400 mL of extraction buffer (50 mM Tris/HCl pH = 8, 250 mM NaCl, 0,1 mM EDTA, 0,1% Triton X-100, 1 mM PMSF) and disrupted using a high pressure homogenizer (Panda NS1001L, GEA Niro Soavi, Italy). The recovered suspension was cooled on ice, 2-mercaptoethanol was added to 20 mM and centrifuged at 10000 rpm, for 30 min at 8 °C (Beckman–Coulter, J-26 XP). Supernatant was properly diluted adding fresh extraction buffer and precipitated adding NaCl to a final concentration of 1.5 M at 37 °C. The aggregated polypeptide particles were separated by centrifugation at 7000 rpm, 37 °C for 30 min. The pellet was re-dissolved in cold water, non-soluble material was discarded after cold centrifugation and solution was precipitated again by NaCl addition and rising temperature to 37 °C. Three ITCs yield pure recombinant protein. After the last temperature-dependent transition cycle the material was lyophilized for long-term storage and a solution 2 mg/mL was prepared to analyze by SDS-PAGE.

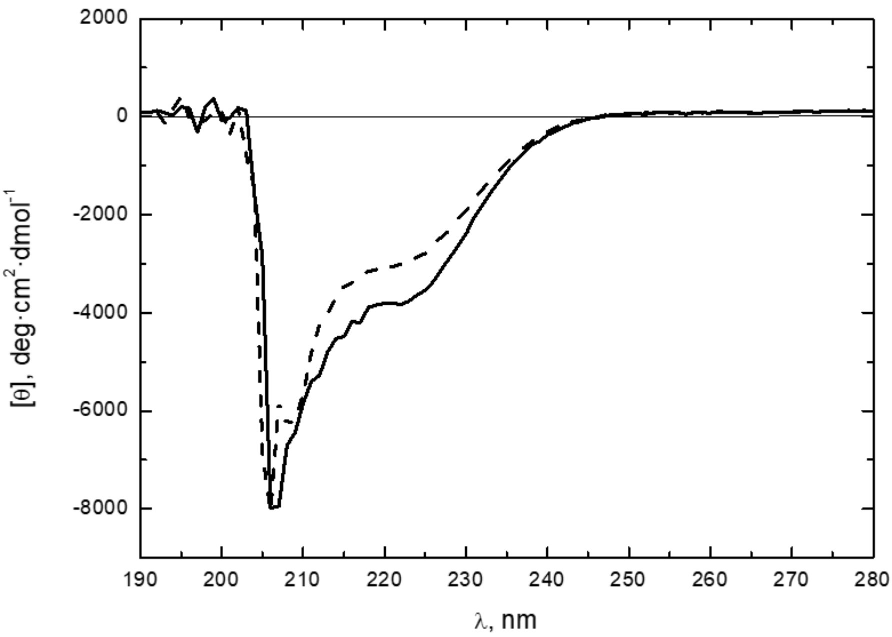

Figure 1S

Circular dichroism (CD) spectra of HIn (black line) compared with that of the HELP biopolymer (dashed line). Spectra were recorded on protein solutions with a concentration of 0.1 mg/mL in NaPi / NaCl (10 mM sodium phosphate/ 0.15M NaCl pH = 6.8) buffer. The CD Spectra were recorded at 25 °C in a 200- to 500-nm thermostatic cell on a Jasco J-710 spectrometer under constant nitrogen flow, and the data were expressed as the mean molar ellipticity [θ] of the residue (mdeg-cm2-dmol-1).

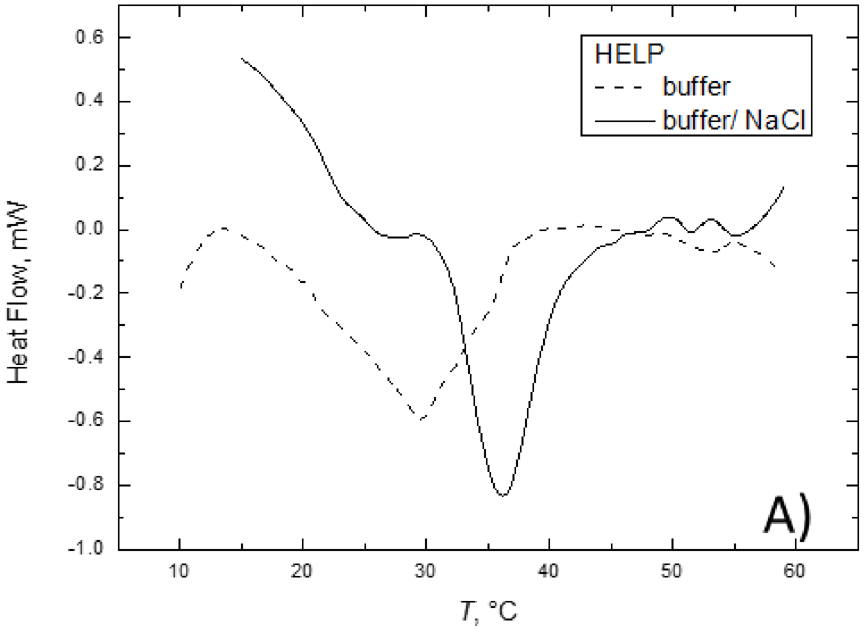

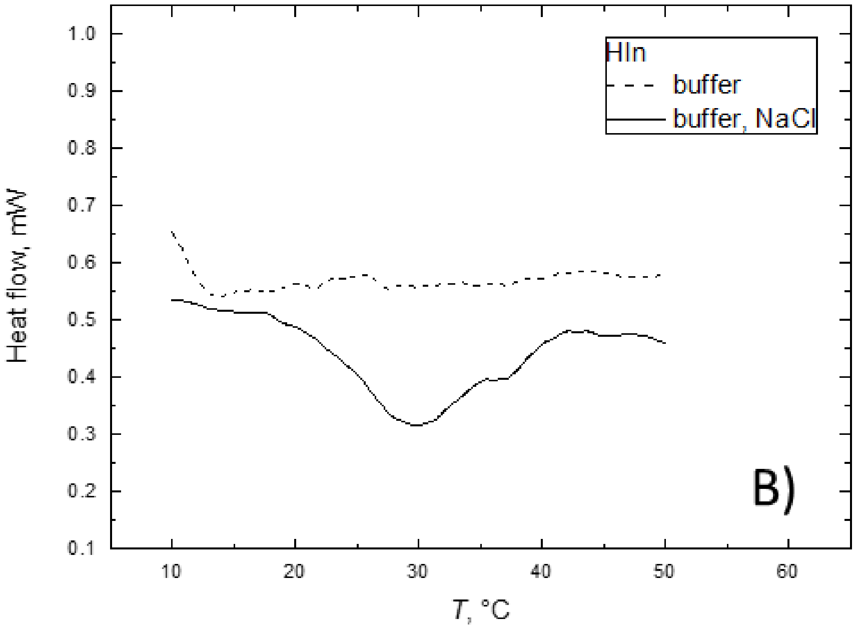

Figure 2S

DSC thermograms of A) HELP and B) HIn biopolymers 4 mg/mL in NaPi (10 mM sodium phosphate pH = 6.8, dotted line) and NaPi / NaCl (10 mM sodium phosphate/ 0.15M NaCl pH = 6.8, black line) buffers.

Stainless steel cells were filled by weight with protein samples and then hermetically sealed and equilibrated for 16 h at 4 °C. The calorimeter was pre-equilibrated at 5°C for 10 min, followed by heating from 5 to 70 °C at a scan rate of 0.5 °C/min. The solvent was used as a reference. The onset and the peak inverse transition temperature (Tt) were determined.

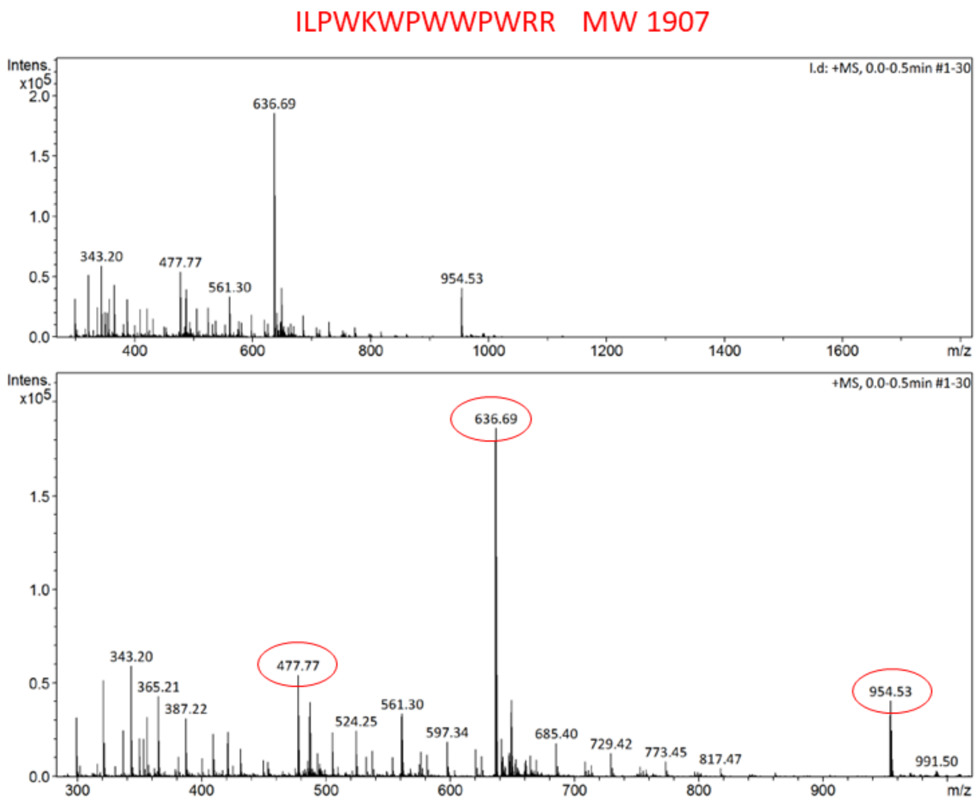

Figure 3S

Electrospray ionization Mass Spectrometry analysis of the indolicidin domain obtained by specific Glu-C cleavage of a 4% HIn matrix. A 150 µL matrix was incubated overnight at 37˚C with 500 µL of 100 mM ammonium bicarbonate buffer pH 8 with 10 ng/µL of Glu-C enzyme. After the reaction, the supernatant was collected, added by 0.1% TFA and injected with a syringe infusion pump at 2 µl/min, scanning at m/z 300/1800. Positive-ion detection was performed at an orifice potential of 75 V.

The peptide masses from your sequence are:

[Theoretical pI: 12.01 / Mw (average mass): 1907.30 / Mw (monoisotopic mass): 1906.03]

mass	position	#MC	peptide sequence
636.3508	1-13	0	ILPWKWPWWPWRR

100.0% of sequence covered (you may modify the input parameters to display also peptides < 500 Da or > 100000000000 Da):

10
ILPWKWPWWP WRR

The peptide masses from your sequence are:

[Theoretical pI: 12.01 / Mw (average mass): 1907.30 / Mw (monoisotopic mass): 1906.03]

mass	position	#MC	peptide sequence
954.0226	1-13	0	ILPWKWPWWPWRR

100.0% of sequence covered (you may modify the input parameters to display also peptides < 500 Da or > 100000000000 Da):

10
ILPWKWPWWP WRR

Table 1S

Theoretical masses of the expected ions predicted by PeptideMass (Expasy Server, https://web.expasy.org/peptide_mass/)
